# Anthropogenic landscapes increase *Campylobacter jejuni* infections in urbanizing banded mongoose (*Mungos mungo*): A one health approach

**DOI:** 10.1371/journal.pntd.0007888

**Published:** 2020-03-17

**Authors:** Sarah Medley, Monica Ponder, Kathleen A. Alexander

**Affiliations:** 1 Department of Fish and Wildlife Conservation, Virginia Tech, Blacksburg, Virginia, United States of America; 2 Chobe Research Institute, Centre for Conservation of African Resources, Animals, Communities, and Land use (CARACAL), Kasane, Botswana; 3 Department of Food Science and Technology, Virginia Tech, Blacksburg, Virginia, United States of America; University of Colorado Health Sciences Center, UNITED STATES

## Abstract

**Background:**

*Campylobacter* is a common, but neglected foodborne-zoonotic pathogen, identified as a growing cause of foodborne disease worldwide. Wildlife and domestic animals are considered important reservoirs, but little is known about pathogen infection dynamics in free-ranging mammalian wildlife particularly in sub-Saharan Africa. In countries like Botswana, there is significant overlap between humans and wildlife, with the human population having one of the highest HIV infection rates in the world, increasing vulnerability to infection.

**Methodology/Principal findings:**

We investigated *Campylobacter* occurrence in archived human fecal samples (children and adults, n = 122, 2011), feces from free-ranging banded mongooses (*Mungos mungo*, n = 201), surface water (n = 70), and river sediment samples (n = 81) collected in 2017 from the Chobe District, northern Botswana. *Campylobacter* spp. was widespread in humans (23.0%, 95% CI 13.9–35.4%), with infections dominantly associated with *C*. *jejuni* (82.1%, n = 28, 95% CI 55.1–94.5%). A small number of patients presented with asymptomatic infections (n = 6). While C*ampylobacter* spp. was rare or absent in environmental samples, over half of sampled mongooses tested positive (56%, 95% CI 45.6–65.4%). Across the urban-wilderness continuum, we found significant differences in *Campylobacter* spp. detection associated with the type of den used by study mongooses. Mongooses utilizing man-made structures as den sites had significantly higher levels of *C*. *jejuni* infection (p = 0.019) than mongooses using natural dens. Conversely, mongooses using natural dens had overall higher levels of detection of *Campylobacter* at the genus level (p = 0.001).

**Conclusions:**

These results suggest that landscape features may have important influences on *Campylobacter* species exposure and transmission dynamics in wildlife. In particular, data suggest that human-modified landscapes may increase C. *jejuni* infection, a primarily human pathogen, in banded mongooses. Pathogen circulation and transmission in urbanizing wildlife reservoirs may increase human vulnerability to infection, findings that may have critical implications for both public and animal health in regions where people live in close proximity to wildlife.

## Author summary

*Campylobacter* infections are increasing worldwide but we still know little about the true burden of disease in the developing world, and even less about the role of wildlife and environmental reservoirs in human exposure and infection. Here we take a one-health approach evaluating *Campylobacter* infections in humans, banded mongooses (*Mungos mungo*), and the environment. We found evidence of widespread infection with *Campylobacter* spp. infecting nearly a quarter of sampled adults and children (23.0%, n = 122, 2011), dominantly with *C*. *jejuni*. Banded mongooses live in close association with humans in the area and over half of the animals sampled were found positive for *Campylobacter* spp. (56%, n = 201, 2017). Infection with *C*. *jejuni* was greater among mongooses utilizing human-made structures as den sites than those using natural dens. These data suggest that wildlife utilization of anthropogenic landscapes may increase *C*. *jejuni* exposure and infection. In turn, pathogen circulation and transmission in urbanizing wildlife reservoirs may increase human vulnerability to infection, particularly impoverished populations, where greater environmental exposures are expected.

## Introduction

*Campylobacter* spp. are considered the most common cause of foodborne infections globally, causing an estimated 96 million cases of diarrheal illness in 2010[1]. The true burden of infection remains uncertain in many developing countries [2, 3], as is the role of wildlife and environmental reservoirs in transmission [4]. Human illnesses are dominantly associated with *C*. *jejuni*, however, other *Campylobacter* species of clinical significance are emerging [5]. Differences identified in clinical presentation and serotype distribution indicate that reservoirs and patterns of illness differ at both the local and regional scale [6]. Risk factors for contracting campylobacterosis in developing countries are associated with environmental exposures, including animal contact, contaminated food, source of drinking water, and sanitation access [7, 8]. Zoonotic transmission of *Campylobacter* is thought to occur dominantly from contact with infected livestock and poultry [9]. Wildlife can also act as reservoirs or amplifying hosts, increasing the number of exposure pathways for *Campylobacter* [10]. The incidence and prevalence of *Campylobacter* infections in humans appears to be growing in both developing and developed countries [4]. Across many countries in sub-Saharan Africa, *Campylobacter* is considered to be endemic with both symptomatic and asymptomatic infections a common occurrence [11, 12].

Genetic evidence suggests that the dominant *Campylobacter* strains circulating are generalists, capable of colonizing both animals and humans, but rarer strains may exist that are adapted to only a single host species [13]. The few studies that were conducted in sub-Saharan African were dominantly focused on bushmeat [16], mammals on game farms [17], and avian species [18]. Detailed studies of *Campylobacter* in free ranging wildlife are lacking.

*Campylobacter spp*. persistence in the environment, particularly in soils and water, may also play a key role in pathogen exposure and transmission to humans. *Campylobacter* can persist through incorporation into biofilms as well as entry into a physiological state referred to as viable but nonculturable (VBNC) where metabolic activity and infectivity is maintained [9, 19]. Strains vary in their ability to survive in the environment with certain strains exhibiting aerotolerance, acid tolerance, and starvation survival adaptations [20]. Viable *Campylobacter* are also able to extend their survival by infecting amoeba and protozoan species, acting as both a reservoir and vector for infection [21]. Our limited understanding of pathogen exposure and transmission dynamics at the human-animal-environmental interface has challenged development of appropriate public and animal health interventions. There is an urgent need, however, to improve our understanding of environmental and animal reservoir dynamics and exposure and transmission pathways, particularly in regions where HIV/AIDS may increase population vulnerability to infection [22].

In northern Botswana, diarrheal disease remains a persistent health challenge affecting adults and children alike, with the causative agent in most cases remaining unidentified [23]. At the same time, HIV/AIDS infection levels are one of the highest in the world [24, 25], leaving a large proportion of the population more susceptible to enteric pathogens. Studies previously conducted in Botswana found that *C*. *jejuni* isolates collected from free-ranging chickens and commercial broiler chickens were closely related to isolates obtained from humans suggesting zoonotic transmission [26]. At the human-wildlife interface, where wildlife diversity and density is high, wildlife reservoirs may also play a crucial role in pathogen transmission to domestic animals and humans, but our understanding of this is limited [14, 15].

Banded mongooses provide an important opportunity to advance our understanding of pathogen transmission and persistence dynamics at the human-wildlife-environmental interface. These small, fossorial animals are able to adapt to human environments, living across both natural and anthropogenically-modified landscapes. They eat primarily invertebrates and small vertebrates, but will rely on human-food resources associated with garbage where this is available. Mongooses also utilize man-made structures as dens if accessible [27]. Pathogenic *Leptospira* spp. has also been detected in this species across land type in our study site [28], as well as, *Mycobacterium mungi*, a novel tuberculosis pathogen, transmitted through olfactory communication behavior [29]. This territorial species provides an important opportunity to investigate the way wildlife may be exposed to *Campylobacter* species across landscape type. In particular, we can begin to investigate how urban landscapes may influence wildlife pathogen exposure and transmission risk, the implications this may have on infection dynamics at the human-wildlife interface, and public and animal health. Our previous research suggests that urban landscapes can have an important influence on banded mongoose behavioral ecology; modifying space use, relaxing territoriality with den sharing, increasing dispersal behaviors, and transmission potential for other pathogens, such as *M*. *mungi*. We hypothesize that urban landscapes may have a similar influence on *Campylobacter* pathogen exposure and infection, particularly environmental exposure to *C*. *jejuni*, a primary human pathogen.

## Methods

### Ethics statement

Human samples were collected from Chobe District health facilities under permit from Ministry of Health (HPSME:13/18/1 Vol. X (878)) and all patient data was anonymized before analysis. Mongoose and environmental samples were collected under permit from the Ministry of Environment, Natural Resources Conservation and Tourism (EWT8/36/4). Approval was also obtained from the Virginia Tech Institutional Review Board (#11–573) and Institutional Animal Care and Use Committee (IACUC, protocol 13-164-FIW) for human and animal work conducted in this study.

### Study site

**Fig 1:**
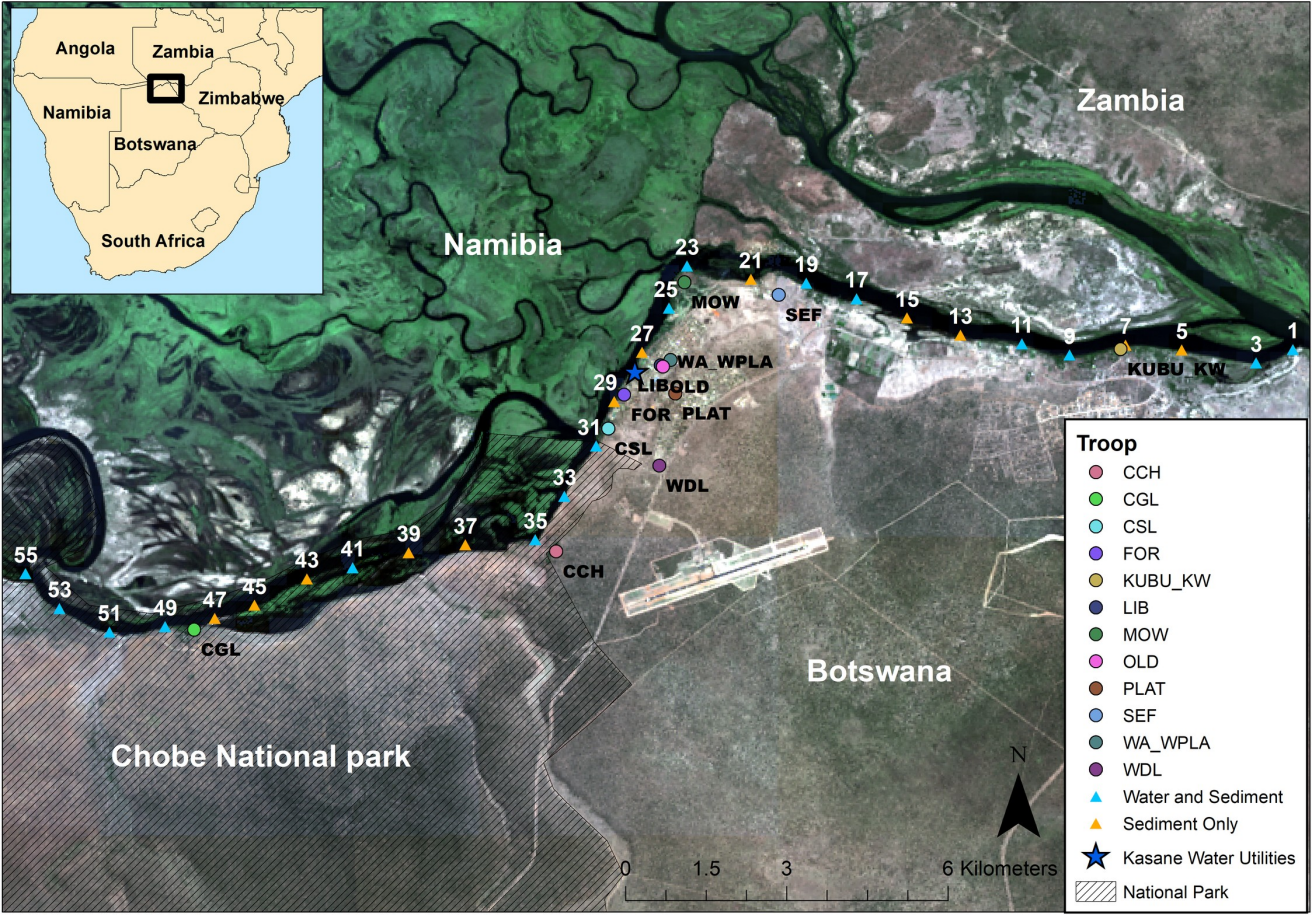
Water, sediment, and banded mongoose (*Mungos mungo*) fecal sample locations along the Chobe River in Chobe District, northern Botswana. Water and sediment samples were collected on established water sampling transect points. Fecal samples were collected at den sites from study banded mongoose troops living in the region. Satellite imagery was obtained for 2013 from the Landsat 8 Operational Land Imager, available from the U.S. Geological Survey (https://earthexplorer.usgs.gov/).

This study was conducted in our long-term study site in northern Botswana along the Chobe River (Fig 1). Chobe National Park is the predominant land area in the district where most of the human population live in the transitioning urban center of Kasane (population 9,008) and the town of Kazungula (population 4,133) [30]. Economic growth is primarily driven by tourism linked to the rich diversity of wildlife resources found in the region. Many tourist lodges surrounding the park concentrate garbage at central, non-animal proof waste sites before periodic disposal at the Kasane landfill. There are no commercial chicken or livestock production systems in the region, although many people keep free-range chickens. The Chobe River supplies domestic water needs, with two conventional water treatment plants treating and distributing the water supply to indoor and outdoor household and public taps [31]. Medical services are subsidized by the government with a small fee charged for health services. There is one laboratory located at the primary hospital in Kasane that analyzes samples collected from government health facilities across the District.

### Sample collection and screening

#### Human

Archived human stool samples originating from both healthy (mandatory employment health certification) and clinically ill patients were collected from District health facilities from August 2011-January 2012. Where available, patient demographic data was collected, including date, age, sex, reason for testing, and any positive culture or virus testing results. Samples were classified by the season in which they were collected (wet season: November-March and dry season: April-October). Samples were collected and stored at -20°C until required for DNA extraction and analysis.

#### Banded mongoose

We regularly monitored 12 banded mongoose troops using VHF radio collars [32]. Banded mongooses use designated communal latrines, where individuals will defecate as a group, providing an opportunity to sample individuals of the troop without duplication. Fecal samples were collected from latrine sites across 12 study troops from July to August 2017 and in January 2019. Briefly, troops were tracked to their denning site the evening prior to fecal collection using radio telemetry [32]. Fecal samples were collected after the mongooses had used the latrine and moved away from the area. A sterile surgical blade was used to sample from the center of the fecal bolus, which was then transferred into a sterilized 1.5 mL Eppendorf tube. The tube was then placed on ice and transported back to the laboratory for immediate DNA extraction.

**Fig 2:**
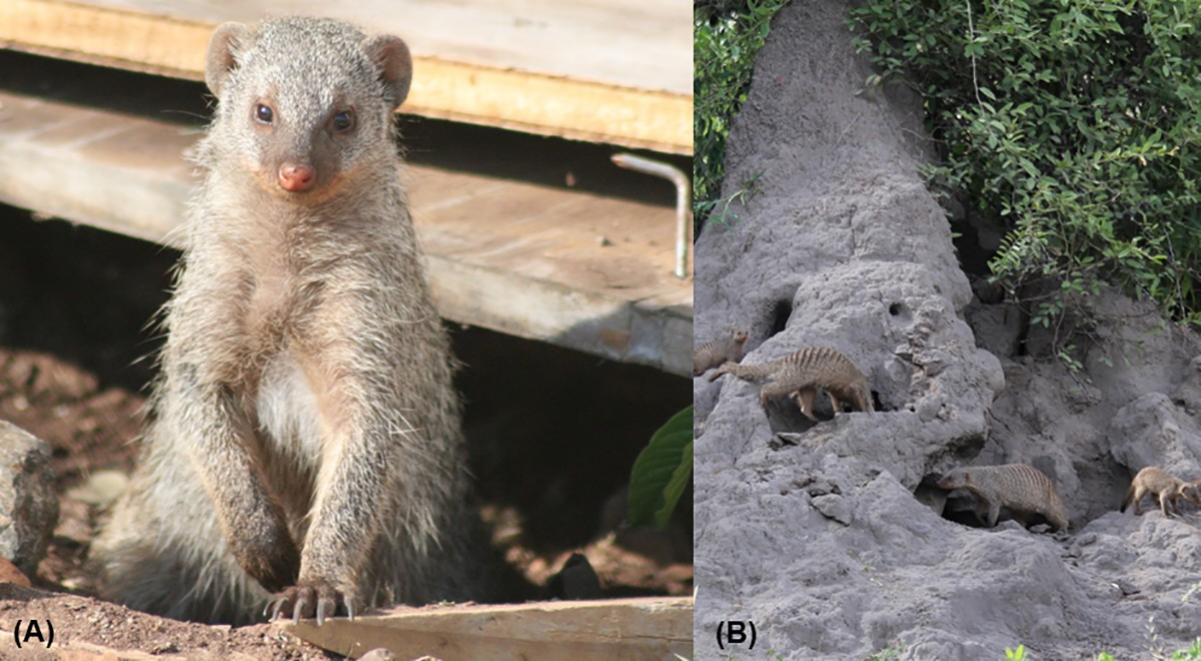
Banded mongooses (*Mungos mungo*) at natural and anthropogenic dens. (A) Banded mongooses emerging from scrap pile (anthropogenic den) Photo credit: Dr. Peter Laver. (B) Banded mongooses at termite mound (natural den) Photo credit: Dr. Claire Sanderson.

Mongoose troop home ranges occurred across five land use types (lodge, national park, residential, undeveloped and urban). Troop land type was identified as the land type dominantly used by the troop over a long-term observation period (2012–2018). These data were also used to calculate the proportion of nights troops were observed in anthropogenic (Fig 2A) or natural (Fig 2B) dens. In contrast to natural den sites, anthropogenic dens were defined as those den sites that were comprised of man-made structures, such as a den site under a foundation, or those associated with human waste (e.g., a pile of scrap metal). Den data was classified into four categories, 1 being lowest proportion of nights in anthropogenic dens and 4 being highest proportion based on quantiles.

#### Water and sediment samples

Water samples were collected and processed using hollow-fiber ultrafiltration as previously published [33]. Hollow-fiber ultrafiltration allows for the simultaneous concentration of bacteria, viruses and parasites from large volumes of water down to approximately 250 mL. From July 2017-August 2017, water (30 L, *n* = 70) and sediment samples (*n* = 81) were collected from 16 locations along the Chobe River. For each sampling event, three to five samples were collected a day over a four-day period with a total of four sampling events. Samples were collected in five-gallon collapsible carboys rinsed with 10% bleach and then distilled water at least 12 hours before sample collection. Once collected, water samples were stored on ice or cold packs and processed within 24 hours. Sediment samples were collected in 50 mL tube at each of the 16 locations during the same week. Environmental samples were kept on cold packs until stored at -20°C until further processed. The final volume of retentate was aliquoted into 50 mL tubes and stored at -20˚C until DNA extraction. A negative filtration control with distilled water was filtered and treated the same as samples.

#### DNA extraction

Fecal and sediment samples were extracted using the PowerSoil DNA Isolation Kit (Qiagen Inc., Germantown, MD) following a bead beating step and normal manufacturer protocols. Retentate obtained from water samples was thawed in cool water for two to three hours after which 10 mL aliquots were spun down and the pellet resuspended in 800 uL of lysis buffer. DNA was then extracted using the PureLinkTM Microbiome DNA Extraction Kit (#A29790, Life Technologies, Carlsbad, CA) following the manufacturer’s soil protocol.

### Detection of *Campylobacter* spp.

The DNA extracts were screened for *Campylobacter* spp. using polymerase chain reaction (PCR) and genus level primers that targeted the 16s rRNA gene (Table 1 [34]). Briefly, the PCR contained 0.5 μM each primer, 1 x HotstarTaq Master Mix (Qiagen, Germantown, MD), 1 μL template DNA, and molecular grade water to reach a 20 μL reaction. All PCR reactions were conducted on MyCycler^™^ thermocycler (Bio-Rad, Hercules, CA). Cycling conditions were set at 95°C for 5 minutes for initial denaturation, 95°C for 30 seconds, primer annealing at 56°C for 30 seconds, and extension at 72°C for 30 seconds for 35 cycles, with a final extension for 4 minutes [35]. Laboratory synthesized fragments (gBlocks, Integrated DNA Technologies Inc., Coralville, IA) containing the same fragment amplified by the primer pairs were used as positive control and a negative (water) control were run with each round of PCR. Negative controls were used to establish lack of non-specific bands of the target molecular weight range. PCR products were visualized on a 1% (w/v) agarose gel stained with ethidium bromide.

**Table 1:** Target genes for PCR amplification.

Species	Target gene	Size (bp)	Primer	Sequence	Primer annealing temp (˚C)	Reference
*Campylobacter* Genus	16S rRNA	816	C412F	GGATGACACTTTTCGGAGC	56	[34]
			C1228R	CATTGTAGCACGTGTGTC		
*C*. *jejuni*	*cj0414*	161	C-1	CAAATAAAGTTAGAGGTAGAATGT	58	[36]
			C-3	CCATAAGCACTAGCTAGCTGAT		
*C*. *coli*	*ask*	502	CC18F	GGTATGATTTCTACAAGCGAG	56	[37]*
			CC519R	ATAAAAGACTATCGTCGCGTG		
*C*. *lari*	*glyA*	251	CLF	TAGAGAGATAGCAAAAGAGA	53	[38]
			CLR	TACACATAATAATCCCACCC		
*C*. *fetus*	*cstA*	764	MG3F	GGTAGCCGCAGCTGCTAAGAT	60	[39]
			MG4R	TAGCTACAATAACGACAACT		
*C*. *hyointestinalis*	23S rRNA	611	HY01F	ATAATCTAGGTGAGAATCCTAG	53	[40]
			HYOFET23SR	GCTTCGCATAGCTAACAT		
*C*. *upsaliensis*	*glyA*	204	CUF	AATTGAAACTCTTGCTATCC	51	[38]
			CUR	TCATACATTTTACCCGAGCT		
*C*. *ureolyticus*	*hsp60*	429	CU-HSP60F	GAAGTAAAAAGAGGAATGGATAAAGAAGC	61	[41]
			CU-HSP60R	CTTCACCTTCAATATCCTCAGCAATAATTAAAAGA		

*CC18F was modified to correct for an error in the sequence [42].

DNA samples that were positive for *Campylobacter* genus were then screened for *C*. *jejuni*, *C*. *coli*, *C*. *lari*, *C*. *fetus*, *C hyointestinalis*, *C*. *upsaliensis*, and *C*. *ureolyticus* using uniplex species-specific PCR assays adapted from Bullman et al. [43] with primer pairs and annealing temperatures listed in Table 1. Each reaction consisted of 1 x HotstarTaq Master Mix (Qiagen, Germantown, MD), 2–3 μL template DNA and 1 μM of each primer. The final volume was adjusted to 25 μL with molecular grade water. Positive gBlocks controls and a negative (water) control were run with each round of PCR. All PCR reactions were conducted on MyCycler^™^ thermocycler (Bio-Rad, Hercules, CA) with one cycle of 95 ˚C for 5 min, followed by 35 cycles of 95 ˚C for 30 seconds, annealing temp for 1 min and 72 ˚C for 1 min and ending with a final extension time at 72 ˚C for 7 min. PCR products were visualized on a 1.5% (w/v) agarose gel stained with ethidium bromide. Samples that were positive using *Campylobacter* genus primers but negative for the seven selected species were classified as unknown *Campylobacter spp*.

### Statistical analysis

Pearson’s Chi-square test was used to evaluate differences in *Campylobacter* and *C*. *jejuni* prevalence by land use and den type. Pearson’s Chi-square test was also used to evaluate the difference in *Campylobacter* prevalence in human samples based on age group and season. Wilson score method was used to estimate 95% confidence intervals with Bonferroni adjustments made for multiple comparisons. Figures were made in R version 3.4.4 and Chi-square tests were performed in JMP Pro 14.

## Results

Almost a quarter of all human samples collected from patients at local health facilities were positive for *Campylobacter* spp. (23.0%, *n* = 122, 95% CI 13.9–35.4%). The mean age of *Campylobacter* infection was 26 (range 6 months-62 years). Children under five years of age (26.5%, *n* = 34, 95% CI 13.4–45.6%) had similar prevalence levels (*P* = 0.645) to that found in older children and adults (22.0%, *n* = 82, 95% CI 12.4–32.3%) (Table 2). At least six samples positive for *Campylobacter* spp. were from adult patients with no gastrointestinal symptoms; four of those patients were positive for *C*. *jejuni* and two could not be identified at the species level. *C*. *jejuni* was the most commonly identified species, representing 82.1% (*n* = 28, 95% CI 55.1–94.5%) of *Campylobacter*-positive samples. Two samples were positive for *C*. *coli*, one sample was positive for *C*. *fetus*, and two samples could not be identified at the species level. We did not detect any human infections with *C*. *lari*, *C*. *upsaliensis*, *C*. *hyointestinalis*, and *C*. *ureolyticus*. There was no variation in infection by season (*P* = 0.153), with 24.3% of dry season samples (*n* = 74, 95% CI 15.0–36.9%) and 29.4% of wet season samples (*n* = 34, 95% CI 15.5–48.6%) positive for *Campylobacter* spp. (Table 2).

**Table 2:** *Campylobacter* genus prevalence in human feces sampled from Chobe District, Botswana by season and age group.

Variables	*n*	*Campylobacter spp*.		*p*^b^
		Prevalence (%)	95% CI^a^	
Season				0.153
Wet	34	24.3	15.5–48.6	
Dry	74	29.4	15.0–36.9	
Age				0.645
Children under 5	34	26.5	13.4–45.6	
Older children and adults	82	22.0	12.4–32.3	

^a^ Wilson score 95% Confidence Interval with Bonferroni adjustment

^b^ Pearson Chi-square *p* values

**Fig 3:**
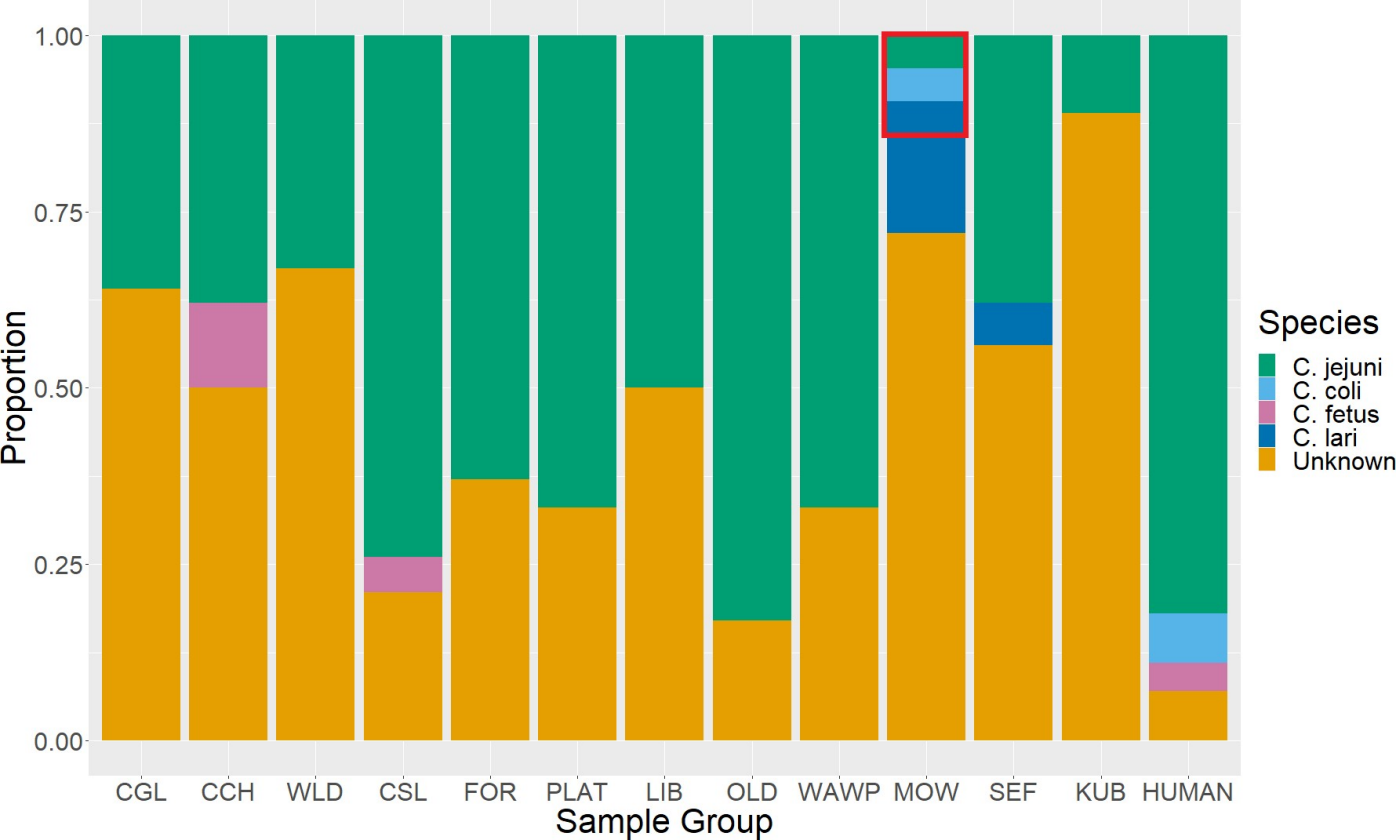
Proportion of samples positive for *C*. *jejuni*, *C*. *coli*, *C*. *fetus*, *C*. *lari*, and unknown species relative to the number of total *Campylobacter*-positive samples in each banded mongoose (*Mungos mungo*) troop and in humans. The highlighted red box represents results obtained from a single mongoose in the MOW troop that was positive for three *Campylobacter* species.

We evaluated 201 fecal samples from 12 mongoose study troops. Of these, 56% (*n =* 201; 95% CI 45.6–65.4%) were found positive for *Campylobacter* genus DNA. Of genus-positive samples, infection with one of the seven tested organisms could only be identified for 52.7% of the screened mongoose fecal samples (*n* = 112, 95% CI 43.5–61.7%). While most were positive for only one of the tested species, one mongoose fecal sample (MOW troop) tested positive for three species (*C*. *jejuni*, *C*. *lari*, and *C*. *coli*). Across sampled mongooses, *C*. *jejuni* was the dominant species identified, accounting for 49.1% (*n* = 112, 95% CI 36.1–62.3%) of *Campylobacter-*positive samples. Indeed, *C*. *jejuni* infections were discovered across nearly a third of all the mongooses sampled in the study system (27.4%, *n =* 201, 95% CI 21.7–34.0%) (Table 3). Other species were sparsely identified, including *C*. *fetus*, *C*. *coli*, and *C*. *lari*. As with the human samples, we did not detect *C*. *upsaliensis*, *C*. *hyointestinalis*, or *C*. *ureolyticus* in sampled mongooses (Fig 3).

**Table 3:** Detection of *Campylobacter* genus and *C*. *jejuni* DNA in banded mongoose (*Mungos mungo*) fecal samples, by troop, land use and den use.

Troop	Land use^a^	Anthropogenic den use^b^	*Campylobacter spp*.			Percent of *Campylobacter* that are *C*. *jejuni*		
			*n*	Prevalence (%)	95% CI^c^	*n*	Prevalence (%)	95% CI
CGL	Lodge	Low	15	93.3	56.5–99.3	14	35.7	11.3–70.7
CCH	Lodge	Medium	23	34.8	14.0–63.7	8	37.5	8.8–79.0
WLD	Residential	High	12	50.0	18.5–82.0	6	33.3	5.8–80.1
CSL	Lodge	High	24	79.2	49.8–93.6	19	73.7	41.2–91.8
FOR	Urban	Very High	11	72.7	32.2–93.7	8	62.5	21.0–91.3
PLAT	Residential	Very High	19	31.6	11.0–63.4	6	66.7	19.8–94.2
LIB	Urban	Medium	6	66.7	19.8–94.1	4	50.0	8.9–91.1
OLD	Urban	Very High	33	36.4	17.4–60.8	12	83.3	42.3–97.2
WAWP	Urban	High	5	60.0	14.6–92.9	3	66.7	12.2–96.6
MOW	Lodge	Low	11	63.6	25.7–89.9	7	14.3	1.4–65.9
SEF	Undeveloped	Medium	21	76.2	45.0–92.6	16	37.5	13.1–70.4
KUBU	Lodge	Medium	21	42.9	18.5–71.3	9	11.1	1.1–58.4
Human	**–**	**–**	122	23.0	13.9–35.4	28	82.1	55.1–94.5
Mongoose	**–**	**–**	201	55.7	45.6–65.4	112	49.1	36.1–62.3

^a^Land use for each troop was classified the same as comparisons made in Table 2

^b^Anthropogenic den use category based on quartiles

^c^95% CI with Bonferroni adjustment visualized below

There was no significant variation in occurrence of *Campylobacter* spp in fecal samples by land use. However, when examining den type (natural or anthropogenic) and *Campylobacter* spp. infections in banded mongooses, we found significant differences in patterns of infection by den type (*p* = 0.001, Table 4). Across troops, 89% (n = 412) of the time mongooses were observed using anthropogenic dens (range 67%-100%). Troops that denned more frequently in natural den sites had an elevated prevalence of *Campylobacter* spp. overall (80.8%, *n =* 26, 95% CI 43.4–95.8%). However, troops that used anthropogenic sites had significantly greater levels *C*. *jejuni* (*P* = 0.019, Table 3).

**Table 4:** Comparison of *Campylobacter* spp. and *C*. *jejuni* prevalence levels in banded mongooses (*Mungos mungo*) by den type and land use. Land use areas used by mongoose troops were classified into five land use types (i.e., lodge, national park, residential, undeveloped and urban) with dominate land type used by each troop calculated from the total number of observations obtained from 2012 to 2018. The proportion of nights spent in anthropogenic dens was calculated for each troop based on the total number of den observations from 2012–2018, and classified into four categories based on quantiles, with 1 being the lowest and 4 being the highest.

Variables	*n*	*Campylobacter spp*.		*P*^b^	*C*. *jejuni*		*p*^b^
		Prevalence (%)	95% CI^a^		Prevalence (%)	95% CI^a^	
Anthropogenic Den Use^c^				**0.001**			**0.019**
1	26	80.8	43.4–95.8		23.1	5.7–59.9	
2	71	52.1	31.1–72.5		16.9	6.1–39.1	
3	41	68.3	39.1–87.9		43.9	20.0–71.1	
4	63	44.4	21.4–64.4		30.2	13.6–54.2	
Land Use^d^				0.025			0.456
Lodge	94	60.6	41.2–77.2		25.5	12.5–45.2	
Residential	31	38.7	14.5–70.2		19.4	4.7–53.7	
Undeveloped	21	76.2	36.0–94.8		28.6	7.1–67.7	
Urban	55	49.1	26.3–72.3		34.6	15.7–59.9	

^a^ Wilson score 95% Confidence Interval with Bonferroni adjustment

^b^ Pearson Chi-square *P* values, critical *p* value was corrected for multiple comparisons using Bonferroni adjustment

^c^ Quantiles, 1 the lowest and 4 the highest proportion of den nights in anthropogenic structures.

^d^ Dominate land type used by troop.

All screened water samples were negative for *Camyplobacter* spp. (*n =* 70, 95% CI 0.0–5.2%). One river sediment sample was positive for *Camplyobacter* spp. (*n* = 81, 0.2–6.7%) during a sampling event on August 1, 2017, but was negative for the seven assessed species.

## Discussion

Across the globe, urbanization and anthropogenic land change is occurring at an unprecedented rate, with uncertain impacts on infectious disease transmission and human and animal health [44]. In this study, we found widespread carriage of *Campylobacter* spp. in both humans and banded mongooses, but environmental detection was either absent or rare. Of critical significance was the identification of increased carriage of the zoonotic pathogen, *C*. *jejuni*, in mongoose troops that used anthropogenic den sites (e.g., mongoose dens associated with man-made structures or materials) in close association with human populations (p = 0.019). In contrast, troops that had a higher proportion of nights in natural den sites had an elevated occurrence of *Campylobacter* spp. overall (p = 0.001). Mongoose troops have varying levels of access to human garbage, human sewage, and human and domestic animal waste [45], with some troops relying more heavily on these anthropogenic resources. Data suggest these urbanizing behaviors in banded mongooses may be associated with increased exposures to human sources of *C*. *jejuni* in the environment.

### Human infections with *Campylobacter*

In sampled humans, *Campylobacter* was detected in 23% of sampled patients with no difference in *Campylobacter* spp. occurrence by age group. Surprisingly, in our study adults and older children were infected as frequently as children under five years of age, findings that diverge from other studies where infection has been dominantly found in young children (reviewed in [46]). This could be due to the increased number of immunocompromised individuals in the population and greater susceptibility to infection related to the high burden of HIV/AIDs infection in the region [47], or because of the inclusion of healthy adults in this study who may have protective immunity to *Campylobacter* and would not normally be screened for infection (reviewed in [48]). Detection of asymptomatic *Campylobacter* spp. infections in the human population indicates *Campylobacter* is endemic in this population, findings observed in other sub-Saharan African countries [49]. The presence of asymptomatic infections has important implications to *Campylobacter* spp. epidemiology, surveillance and risk assessment, complicating public health prevention and control strategies [48].

The majority of *Campylobacter*-positive human samples (82.1%) were *C*. *jejuni*, which is similar to previous reports for southern Botswana (94.7%, 95% CI 75.4–99.1%) [26]. The nature of environmental reservoirs may influence exposure and transmission of *Campylobacter* species with *C*. *jejuni* appearing to have a greater survival in the environment than other species (reviewed in [9]). Although seasonality has been a prominent feature of *Campylobacter* infections in western countries [50], there was no difference in *Campylobacter* occurrence by season in this study. A lack of seasonality in *Campylobacter* spp. has also been observed in other developing countries [51], and may be related to warmer ambient temperatures in tropical regions [52]. Human samples were not collected over the same sampling time as mongoose and environmental samples which limits direct comparison, however, these results provide evidence that *Campylobacter* spp. infections occur in this population, and in 2011 were widespread in patients presenting to local health facilities.

### *Campylobacter* spp. presence in banded mongooses

Over half of the mongooses sampled in this study tested positive for *Campylobacter* spp. which is higher than any other surveyed mammalian species [53–55], and some avian hosts [14, 18]. One mongoose fecal sample (MOW troop) tested positive for three *Campylobacter* species (*C*. *jejuni*, *C*. *lari*, and *C*. *coli*). *C*. *jejuni* was the dominant species identified, infecting nearly a third of all mongooses sampled in the study (27.4%, *n =* 201, 95% CI 21.7–34.0%, Table 3). *Campylobacter* species detection was similar to that observed in humans from the region.

This is the first report of *Campylobacter* infection in banded mongooses and the only report of this pathogen in wildlife in Botswana, findings that have clear implication to both human and animal health given the propensity for banded mongooses to adapt to urbanizing environments. *Campylobacter* spp. has previously has been found in the small Indian mongoose (*Herpestes auropunctatus*), a predominantly solitary species, but at significantly lower levels (2.4–9%) [56, 57] then that found in banded mongooses (56%, 95% CI 46–65%). A number of banded mongoose behaviors may contribute to observed elevations in infection. Banded mongooses, for example, are known to scavenge in human waste, a risk factor for *Campylobacter* carriage in wild birds [58]. Mongooses will forage for insects in the feces of other species, in particular large ruminants [59]. They are also a fossorial species, denning in the ground and foraging in soil [60], potentially increasing exposure to soil-associated *Campylobacter*. Banded mongooses are also highly social and behaviors, such as allogrooming, anogenital inspection, and scent marking with feces, in association with olfactory communication behaviors, may also increase microbial exposure and transmission within mongoose social networks [61]. Den sharing by multiple troops can also occur in urban and lodge environments [62]. Differentiation in infection status and species by behavior and ecological niche have been observed in bird species, with bird species that foraged at ground level having higher *Campylobacter* prevalence than aerial or arboreal species [63].

### Environmental samples

In this study, only one sediment sample were positive for *Campylobacter* spp. and we were unable to detect the organism in water samples. False-negatives are common and have occurred even when *Campylobacter* is implicated in large waterborne outbreaks [64]. Environmental *Campylobacter* spp. can be difficult to detect using PCR as the organism is likely present in low numbers compared to more abundant environmental microbes and there is potential for high concentration of PCR inhibitors [65]. Despite detection limitations, *Campylobacter* spp. have been found in a wide variety of environmental samples in other studies, including biofilms on rock, wood, water, and sediment in riverine systems [66], and have been connected to multiple waterborne outbreaks from community water supplies contaminated after chlorination failure, heavy rainfall, or sewage intrusion (reviewed in [67]). The one positive sediment sample in this study was located at a popular community recreation spot downstream of an area where wastewater treatment effluent is known to enter the Chobe River. The overall low incidence in water and sediment samples in this study suggests that waterborne transmission between humans and wildlife is less likely than direct contact with anthropogenic wastes but does not rule out sporadic cases.

## Conclusion

Zoonotic pathogens such as *Campylobacter* are on the rise across the globe in vulnerable populations [68]. There is a pressing need to improve our understanding of *Campylobacter* transmission dynamics at the human-domestic animal-wildlife-environment interface and the manner in which landscape transformation may influence human and animal exposure and infection dynamics. Variation in *Campylobacter* spp. infection patterns across banded mongoose troops suggests that human-transformed landscapes may influence pathogen exposure and transmission potential. Here, banded mongoose territorial behavior provides a unique opportunity to evaluate the potential for landscape features to influence pathogen transmission and persistence dynamics, information essential for prevention and infection control. Development of effective public health policy will require that we refine our understanding of human-animal-environmental couplings in pathogen transmission and persistence dynamics.
